# Multiple small intestinal metastatic tumours: from basics to advanced testing

**DOI:** 10.1093/omcr/omaf308

**Published:** 2026-02-18

**Authors:** Sayed A Almahari, Abed M Zaitoun, Mina Fouad, Dileep N Lobo

**Affiliations:** Department of Cellular Pathology, Nottingham University Hospitals NHS Trust, Queen’s Medical Centre, Derby Road, Nottingham NG7 2UH, Nottinghamshire, United Kingdom; Department of Cellular Pathology, Nottingham University Hospitals NHS Trust, Queen’s Medical Centre, Derby Road, Nottingham NG7 2UH, Nottinghamshire, United Kingdom; Nottingham Digestive Diseases Centre, Division of Translational Medical Sciences, School of Medicine, University of Nottingham, Queen’s Medical Centre, Derby Road, Nottingham NG7 2UH, Nottinghamshire, United Kingdom; National Institute for Health Research Nottingham Biomedical Research Centre, Nottingham University Hospitals NHS Trust and University of Nottingham, Queen’s Medical Centre, Nottingham, United Kingdom; Nottingham Digestive Diseases Centre, Division of Translational Medical Sciences, School of Medicine, University of Nottingham, Queen’s Medical Centre, Derby Road, Nottingham NG7 2UH, Nottinghamshire, United Kingdom; National Institute for Health Research Nottingham Biomedical Research Centre, Nottingham University Hospitals NHS Trust and University of Nottingham, Queen’s Medical Centre, Nottingham, United Kingdom; Department of Surgery, Nottingham University Hospitals NHS Trust, Queen’s Medical Centre, Derby Road, Nottingham, NG7 2UH, Nottinghamshire, United Kingdom; Nottingham Digestive Diseases Centre, Division of Translational Medical Sciences, School of Medicine, University of Nottingham, Queen’s Medical Centre, Derby Road, Nottingham NG7 2UH, Nottinghamshire, United Kingdom; National Institute for Health Research Nottingham Biomedical Research Centre, Nottingham University Hospitals NHS Trust and University of Nottingham, Queen’s Medical Centre, Nottingham, United Kingdom; Department of Surgery, Nottingham University Hospitals NHS Trust, Queen’s Medical Centre, Derby Road, Nottingham, NG7 2UH, Nottinghamshire, United Kingdom; MRC Versus Arthritis Centre for Musculoskeletal Ageing Research, School of Life Sciences, University of Nottingham, Queen’s Medical Centre, Derby Road, Nottingham NG7 2UH, Nottinghamshire, United Kingdom; Division of Surgery, Perelman School of Medicine, University of Pennsylvania, 3400 Spruce Street, Philadelphia, PA 19104, United States

**Keywords:** small intestinal neoplasms, lung adenocarcinoma, dedifferentiated carcinoma, gastrointestinal bleeding, molecular diagnostics, antigenic drift

## Abstract

We report the case of a patient with 13 metastatic small bowel tumours presenting with upper gastrointestinal bleeding. He had a video-assisted thoracoscopic left upper lobectomy a year previously for a lung adenocarcinoma. Histological findings of the resected small bowel confirmed a poorly differentiated anaplastic adenocarcinoma (pT3 N1 Mx). Molecular testing on the tumour showed that it harboured KRAS c.182A > T (p.Gln61Leu) mutation, which was similar to the mutation reported on the primary lung adenocarcinoma. Based on this he received adjuvant chemotherapy and immunotherapy (carboplatin, pemetrexed and pembrolizumab) and a CT scan performed two months after the small bowel resection demonstrated no evidence of tumour recurrence. He, however, developed chemotherapy-related nausea and vomiting, and with further deterioration was placed on a palliative care pathway and died 3 months after the bowel resection. This case highlights the role of integrating histopathology, immunohistochemistry, and molecular genetics in distinguishing metastatic from primary small intestinal adenocarcinomas.

## Introduction

Although the small intestine constitutes approximately 75% of the length of the gastrointestinal (GI) tract, neoplasms in this region are relatively uncommon, accounting for only 3%–6% of all tumours in the GI tract [1, 2]. Primary small intestinal malignancies are particularly rare, although their incidence has shown a gradual increase over the past four decades [1, 3].

The most frequent primary tumours of the small intestine include carcinoid tumours (~33%), adenocarcinomas (~30%), lymphomas (~16%), gastrointestinal stromal tumours (GISTs) (7%) and other rare tumours (13%) [2]. Anatomically, adenocarcinomas predominantly occur in the duodenum, while both adenocarcinomas and GISTs are seen in the jejunum. Carcinoid tumours and lymphomas are more often found in the ileum [2, 4].

Metastatic tumours of the small intestine most commonly originate from melanomas, and lung, breast, colorectal, renal and ovarian carcinomas. In clinical practice, metastatic tumours of the small intestine are encountered more frequently than primary ones. The possibility of small intestinal metastases should be considered in patients with a known primary malignancy who develop new gastrointestinal symptoms [3, 5].

These lesions may present radiologically and intraoperatively as circumferential asymmetric wall thickening, intraluminal polypoid or fungating masses, or exophytic growths. They are often multifocal. Clinically, patients may present with gastrointestinal bleeding, bowel obstruction, perforation (reported in up to 42% of cases), or nonspecific abdominal pain [5].

Here, we report a diagnostically challenging case, both clinically and pathologically. A combination of basic histological stains and advanced molecular techniques was used to reach the final diagnosis.

## Case presentation

A 69-year-old man with a history of chronic obstructive pulmonary disease and lung adenocarcinoma, previously treated with a video-assisted thoracoscopic left upper lobectomy and lymph node dissection a year ago, presented with upper gastrointestinal bleeding manifested by melaena and severe anaemia (haemoglobin 62 g/l). He required multiple blood transfusions and intravenous iron replacement.

**Figure 1:**
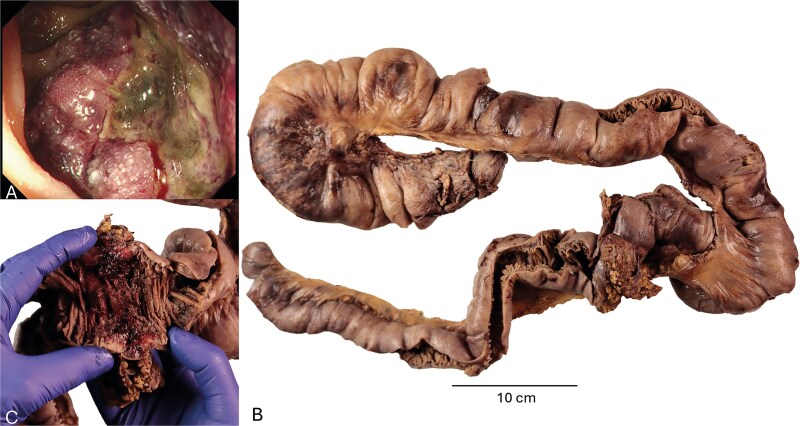
Endoscopic view of a polypoidal tumour with bleeding, ulceration and necrosis in the proximal jejunum (A). Multiple tumour deposits were seen along the 1.2 m length of the resected small intestine (B) with ulceration, haemorrhage and necrosis on the luminal aspect of one of the tumours.

Oesophagogastroduodenoscopy revealed a 40 mm cratered ulcer in the duodenum (D4)/proximal jejunum (Fig. 1A), and biopsies showed intestinal mucosa infiltrated by discohesive malignant cells with hyperchromatic, pleomorphic nuclei and abundant eosinophilic cytoplasm. Immunohistochemistry was negative for CK7, CK20, TTF1, CDX2, SOX10, S100, synaptophysin, and chromogranin A. All mismatch repair proteins were retained, indicating mismatch repair proficiency. The findings were consistent with a poorly differentiated carcinoma, and further evaluation was advised to determine whether the lesion was primary or metastatic.

**Figure 2:**
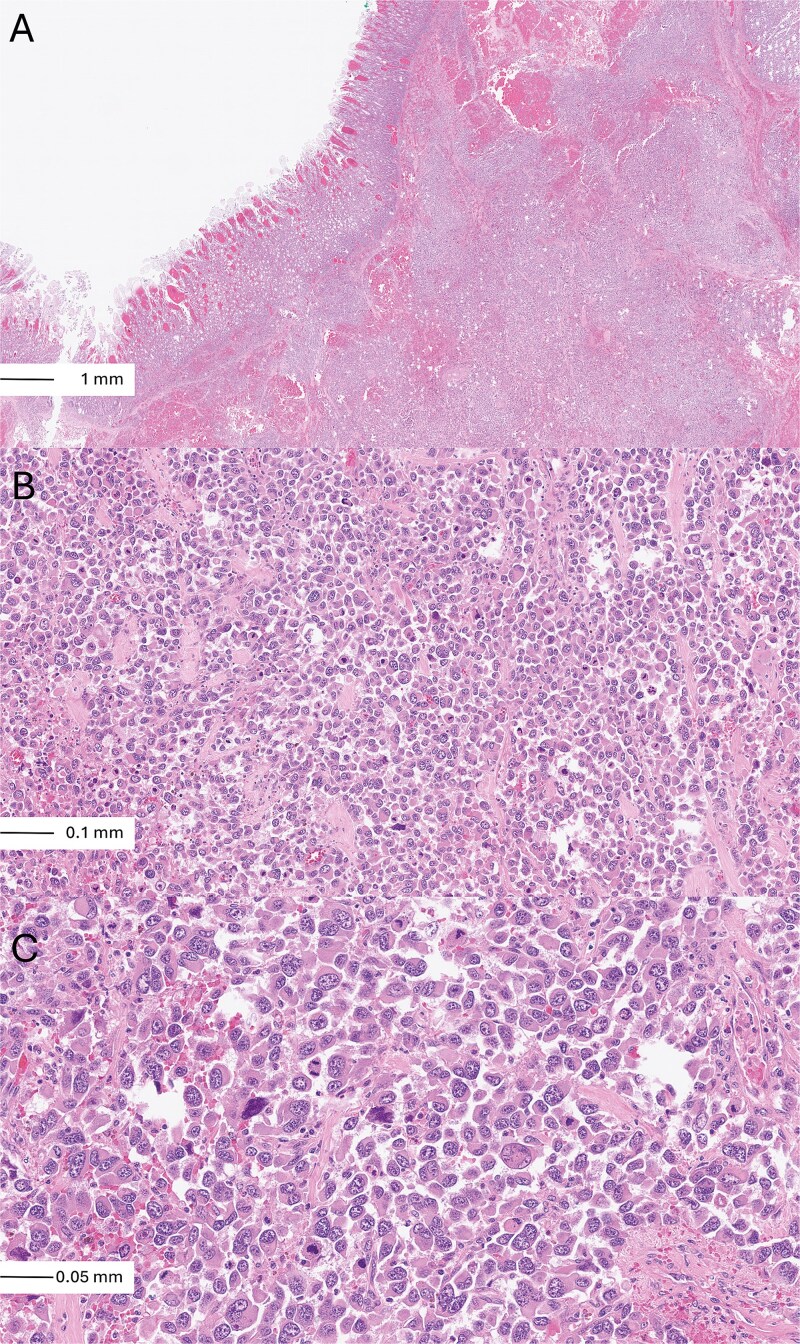
Histological examination of the tumour: Low power view [haematoxylin and eosin (H & E), ×5] showing sheets of malignant cells with areas of necrosis and haemorrhage (A). Intermediate power (H & E, ×20 B) and high power (H & E, ×40 C) magnification views revealed a poorly differentiated anaplastic looking adenocarcinoma. The tumour was composed of sheets of highly malignant looking cells with small rim of eosinophilic cytoplasm, severely pleomorphic nuclei, irregular nuclear membranes and prominent nucleoli.

**Figure 3:**
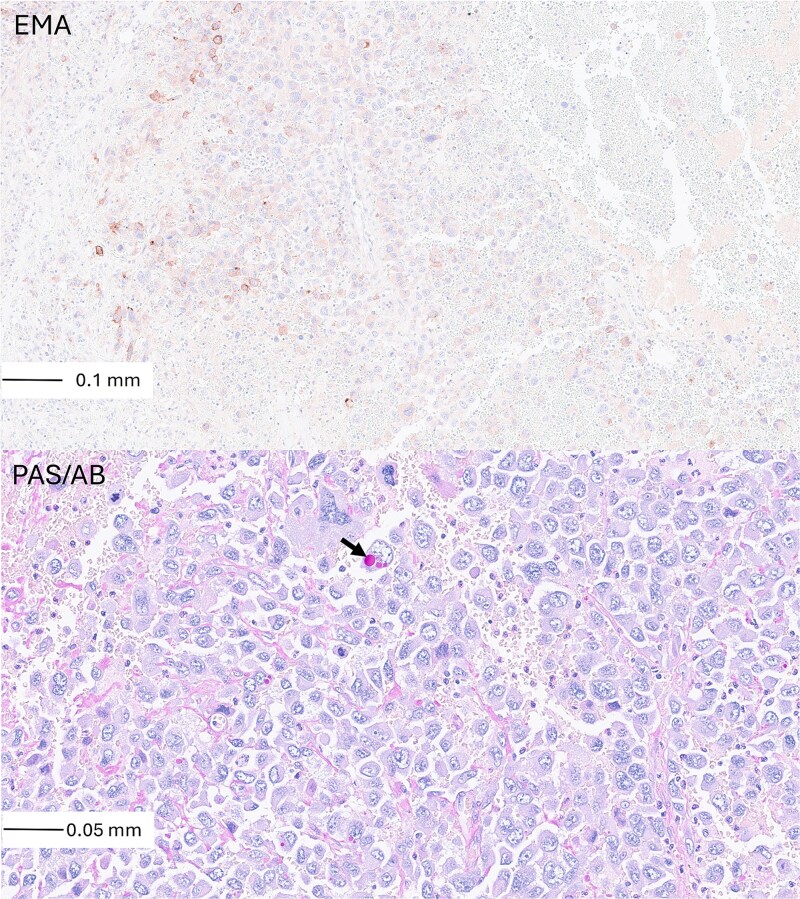
Epithelial membrane antigen (EMA) immunostain showed focal membranous positivity in tumour cells, consistent with epithelial origin (×20, top). Diastase periodic acid-Schiff/Alcian blue (PAS/AB) stain demonstrated variable cytoplasmic mucin content (arrow), supporting adenocarcinomatous differentiation (×40, bottom).

Despite supportive measures, the patient remained transfusion-dependent and underwent urgent exploratory laparotomy. Multiple tumours extending from the proximal jejunum to the proximal ileum were identified, necessitating extensive small bowel resection with a side-to-side jejuno-ileal anastomosis. The postoperative course in the high-dependency unit was uneventful, and the patient was discharged once medically and surgically stable.

Macroscopically, the resected specimen showed thirteen polypoid and ulcerated nodules up to 120 mm in size, extending from the proximal jejunum to the proximal ileum (Fig. 1B and C). The lesions demonstrated extensive surface ulceration, haemorrhage, and areas of necrosis. Some of the tumours infiltrated through the full thickness of the bowel wall with serosal involvement and extension into the omental fat.

Histologically, the neoplasms comprised poorly differentiated adenocarcinomatous cells with anaplastic morphology, forming solid sheets of markedly pleomorphic malignant cells with irregular nuclear membranes, vesicular chromatin, prominent nucleoli, and frequent atypical mitotic figures (Fig. 2). Multinucleated tumour giant cells were present, with extensive necrosis and haemorrhage. Multiple foci of lymphovascular invasion were identified. The nearest tumour was 3.3 mm from the mesenteric resection margin. Seven mesenteric lymph nodes were retrieved, four of which contained metastatic carcinoma.

Immunohistochemistry showed focal epithelial membrane antigen (EMA) positivity (Fig. 3A), while all other epithelial markers (Pan-CK, MNF116, CK7, MUC2, BerEP4, CEA) were negative. Markers for other lineages, including hepatocellular (glypican-3, HepPar-1), lymphoid (CD45, CD3, CD20, CD138), neuroendocrine (synaptophysin, chromogranin A), trophoblastic (β-hCG, P57), muscle (SMA, desmin, MyoD1, myogenin), GIST (CD117, DOG1), melanocytic (S100, HMB45, SOX10, Melan-A), pulmonary (TTF-1, napsin A, p63), breast/urothelial (GATA-3), sarcomatous/vascular (CD99, CD31, CD34, Factor VIII), and mesothelial tumours (WT1) were negative.

Focal intracytoplasmic mucin was demonstrated on diastase periodic acid–Schiff (PAS)/Alcian blue stain, supporting adenocarcinoma differentiation (Fig. 3B). Perls’ stain showed interstitial iron deposition, and Masson–Fontana stain was negative for melanin pigment.

The findings confirmed a poorly differentiated anaplastic adenocarcinoma (pT3 N1 Mx) with four positive lymph nodes, consistent with metastatic lung adenocarcinoma. Complete (R0) resection was achieved.

Further molecular testing on the tumour showed that it harboured KRAS c.182A > T (p.Gln61Leu) mutation, which was similar to the mutation reported on the primary lung adenocarcinoma. Multidisciplinary team review corroborated the diagnosis. The patient was reviewed at 4-week follow-up and was clinically well. He subsequently commenced adjuvant chemotherapy and immunotherapy (carboplatin, pemetrexed and pembrolizumab) under the care of the medical oncology team. Computed tomography performed two months after the small bowel resection demonstrated no evidence of tumour recurrence. The following month, the patient was admitted under the acute oncology service with chemotherapy-related nausea and vomiting. He subsequently deteriorated clinically, was managed with a palliative approach focused on comfort, and died three weeks later.

## Discussion

Distinguishing between a primary and a metastatic lesion is essential (Table 1) [5–8], particularly in patients with a history of malignancy, as this distinction significantly impacts both the diagnostic approach and therapeutic strategy.

**Table 1:** Features differentiating primary from metastatic small intestinal tumours tumours [5–8].

Features	Primary tumours	Metastatic tumours
Number of lesions	Usually solitary	Often multiple
Distribution	Type-specific predilection	Random in all parts of small bowel, but jejunum on of the common sites
Growth pattern	Varies by type (annular for adenocarcinoma)	Often fungating, polypoid, intraluminal masses
Morphology on computed tomography	Type-specific patterns (mesenteric stranding in carcinoid)	Wall thickening, enhancing masses
Clinical presentation	Often indolent with chronic symptoms	Acute manifestations: bleeding (27%), perforation (42%), obstruction (20%)
Immunohistochemistry	Type-specific lineage markers	Reflects primary tumour
Associated findings	Local disease, regional lymphadenopathy	Evidence of primary tumour elsewhere, often with additional metastatic sites

In the present case, the multiplicity and gross morphology of the lesions were atypical for a primary small bowel adenocarcinoma and raised the concern of metastatic disease, especially in the presence of a previous lung primary. Typically, primary small intestinal adenocarcinomas are solitary and most often arise in the duodenum, whereas multifocality, particularly in the jejunum, is more characteristic of haematogenous metastases [7].

Histologically, the tumours displayed high-grade features, comprising solid sheets of anaplastic cells with prominent pleomorphism, tumour giant cells, abundant mitotic activity (including atypical figures), and extensive necrosis. These findings could be compatible with both poorly differentiated primary adenocarcinomas and a metastatic high-grade adenocarcinoma. However, the absence of an associated precursor lesion, such as dysplasia or adenomatous change, further suggested a metastatic process [9].

Immunohistochemistry posed a diagnostic challenge. The tumour cells were negative for lineage-specific markers across epithelial, lymphoid, neuroendocrine, melanocytic, hepatocellular, and mesothelial lineages. Notably, all conventional pulmonary markers, including TTF-1 and napsin A, were negative. Only focal EMA positivity was observed, presenting a dilemma regarding the site of origin. In such contexts, reliance on immunohistochemistry alone may be misleading, particularly when dealing with poorly differentiated or mucinous neoplasms, which are known to exhibit antigenic drift and loss of marker expression during metastatic progression [10].

This phenotypic aberration is especially well documented in pulmonary invasive mucinous adenocarcinomas, which often lack TTF-1 and napsin A expression and may histologically mimic gastrointestinal primaries [11]. Furthermore, the detection of intracytoplasmic mucin on PAS/Alcian blue staining supported glandular differentiation but remained non-specific.

A pivotal finding in this case was the identification of a shared somatic KRAS c.182A > T (p.Gln61Leu) mutation in both the resected small bowel tumours and the primary lung adenocarcinoma diagnosed one year earlier. This molecular concordance provided definitive evidence of clonal origin and confirmed that the jejunal tumours were indeed metastases. KRAS mutations are not unique to lung cancer and also occur frequently in colorectal and pancreatic carcinomas. However, the precise mutation match in two anatomically distinct tumours from the same patient strongly supported metastatic spread rather than separate primaries [12, 13].

This case underscores the critical role of molecular profiling in resolving ambiguous immunophenotypic findings, particularly when conventional immunohistochemistry is inconclusive. It also highlights the enduring diagnostic utility of traditional pathological techniques, such as special stains, in the subclassification of poorly differentiated tumours. In such scenarios, comprehensive integration of clinical history, morphological assessment, immunohistochemistry, and molecular data is essential to establish an accurate diagnosis.

Clinically, recognising this as metastatic lung adenocarcinoma rather than a primary small intestinal malignancy drastically altered management. Surgical resection was performed for symptomatic control of bleeding, and systemic chemotherapy and immunotherapy (carboplatin, pemetrexed and pembrolizumab) were commenced. Misclassification could have led to inappropriate use of GI-specific chemotherapy.

From a broader perspective, this case illustrates several important teaching points. Multifocal small intestinal tumours in a patient with a history of carcinoma elsewhere should prompt immediate suspicion for metastases. Immunohistochemical profiles may vary or be lost in metastatic lesions, particularly in mucinous or poorly differentiated tumours. While EMA and PAS stains can aid in confirming epithelial and mucinous differentiation, respectively, they lack site specificity. Molecular analysis, particularly when demonstrating shared mutations, is invaluable in confirming clonality. Ultimately, a multidisciplinary approach remains the cornerstone in the evaluation of such diagnostically challenging cases.

## Conclusion

In conclusion, this case highlights the critical role of integrating histopathology, immunohistochemistry, and molecular genetics in distinguishing metastatic from primary small intestinal adenocarcinomas. Awareness of immunophenotypic variability, especially in mucinous pulmonary tumours, and judicious use of molecular tools can prevent diagnostic pitfalls and guide optimal patient care.

## Data sharing

Case report, no additional data to share.
